# A Precise Bicoid Gradient Is Nonessential during Cycles 11–13 for Precise Patterning in the *Drosophila* Blastoderm

**DOI:** 10.1371/journal.pone.0003651

**Published:** 2008-11-07

**Authors:** Elena M. Lucchetta, Meghan E. Vincent, Rustem F. Ismagilov

**Affiliations:** Department of Chemistry and Institute for Biophysical Dynamics, The University of Chicago, Chicago, Illinois, United States of America; University of Nottingham, United Kingdom

## Abstract

**Background:**

During development, embryos decode maternal morphogen inputs into highly precise zygotic gene expression. The discovery of the morphogen Bicoid and its profound effect on developmental programming in the *Drosophila* embryo has been a cornerstone in understanding the decoding of maternal inputs. Bicoid has been described as a classical morphogen that forms a concentration gradient along the antero-posterior axis of the embryo by diffusion and initiates expression of target genes in a concentration-dependent manner in the syncytial blastoderm. Recent work has emphasized the stability of the Bicoid gradient as a function of egg length and the role of nuclear dynamics in maintaining the Bicoid gradient. Bicoid and nuclear dynamics were observed but not modulated under the ideal conditions used previously. Therefore, it has not been tested explicitly whether a temporally stable Bicoid gradient prior to cellularization is required for precise patterning.

**Principal Findings:**

Here, we modulate both nuclear dynamics and the Bicoid gradient using laminar flows of different temperature in a microfluidic device to determine if stability of the Bicoid gradient prior to cellularization is essential for precise patterning. Dramatic motion of both cytoplasm and nuclei was observed prior to cellularization, and the Bicoid gradient was disrupted by nuclear motion and was highly abnormal as a function of egg length. Despite an abnormal Bicoid gradient during cycles 11–13, Even-skipped patterning in these embryos remained precise.

**Conclusions:**

These results indicate that the stability of the Bicoid gradient as a function of egg length is nonessential during syncytial blastoderm stages. Further, presumably no gradient formed by simple diffusion on the scale of egg length could be responsible for the robust antero-posterior patterning observed, as severe cytoplasmic and nuclear motion would disrupt such a gradient. Additional mechanisms for how the embryo could sense its dimensions and interpret the Bicoid gradient are discussed.

## Introduction

Remarkably, embryos produce precise patterns of protein expression in spite of perturbations such as differences in gene dosage and uniform changes in the temperature at which they develop. The mystery of how embryos compensate for these perturbations has long fascinated researchers across disciplines.

The Bicoid (Bcd) protein in *Drosophila* embryos has been used as a model to determine how embryos translate a maternal input into robust patterns of zygotic gene expression. Bcd protein is translated from maternally provided mRNA and is thought to diffuse away from its source of production at the anterior pole of the embryo and set up a concentration gradient along the antero-posterior axis of the embryo [1]–[6]. Bcd has been described as a classical morphogen that orchestrates patterning along the antero-posterior axis of the embryo in a concentration-dependent manner in the syncytial blastoderm [1], [2]. Bcd regulates expression of zygotic gap genes, and expression of Bcd and the protein products of the gap genes *hunchback* (*hb*), *krűppel* (*kr*), *giant* (*gt*), *knirps* (*kni*), and *tailless* (*tll*) collectively determine the spatial position of the seven stripes of the pair-rule gene *even-skipped* (*eve*), a classical fate marker [7]. When shifts in the Bcd gradient have been induced by altering the copy number of maternal *bcd* genes, embryos have had corresponding shifts in downstream Even-skipped (Eve) expression, although these shifts were not as great as was predicted by a simple gradient model [3]. Embryos compensated for these shifts in Eve expression by apoptosis after gastrulation [3]. The ability of embryos to compensate for varying amounts of Bcd demonstrates robustness in later developmental stages.

Proper Bcd dosage is an essential input for the proper output of Eve patterning during cycle 14 [3]. Is stability in the shape of the Bcd gradient required for proper patterning? Recent work measured the intensity profile *in-vivo* of a Bcd-eGFP fusion protein and showed that the Bcd gradient is stable to within 10% of egg length [8]. Bcd is localized in nuclei, and nuclei have been proposed to function in maintaining a stable Bcd gradient by contributing to the degradation of Bcd [8]. Because Bcd displayed the same level of precision as one of its outputs, Hunchback (Hb) [9], it may appear that the embryo does not require mechanisms to correct for variations in the Bcd gradient prior to gastrulation [10]. However, the role of stability of the Bcd gradient in the context of patterning has not been tested explicitly, as the Bcd gradient was observed but not modulated. Additionally, although the Bcd gradient has been shown to vary with temperature, the Hb pattern did not vary under the same conditions [11]. These observations suggest that the embryo may be robust to variations in maternal gene expression at earlier stages of development than previously thought.

We previously showed that patterning of both Hb and Eve is normal during cycle 14 in embryos exposed to the environmental perturbation of a temperature step [13]. However, in these embryos, nuclear density was highly disrupted prior to cycle 14, the time period during which Bcd is presumably activating its target genes that, together with Bcd, give rise to the refined pattern of Eve expression. Data was obtained from nuclear staining after fixation at single time points in development [13], leaving the mechanism for how nuclei compensate for differences in density by cycle 14 unclear. Because Bcd is localized in nuclei, disruption of nuclear density and correction of these abnormalities would presumably affect the Bcd gradient.

Pair-rule patterning translates early maternal inputs into later cell fate determination. In order to determine if stability of the Bcd gradient prior to cellularization is essential for pair-rule patterning, here we use a microfluidic device to perturb development with a temperature step [12], [13] and modulate both nuclear divisions and the Bcd gradient. We show that nuclei in embryos exposed to the temperature step undergo severe oscillatory motion along the antero-posterior axis, and the Bcd protein, which is localized within nuclei during nuclear division cycles 10–14, is also perturbed by this motion. However, while the embryo ultimately corrects for abnormalities in nuclear density, the Bcd gradient remains abnormal through cycle 14 to gastrulation. Surprisingly, Eve patterning in embryos with abnormal Bcd remains precise.

## Results

To determine the dynamics of the disruption of nuclear density and the mechanism by which nuclear density is corrected in embryos exposed to a temperature step, we observed in real-time both cytoplasmic motion and nuclear density in embryos while they were exposed to the temperature step in a microfluidic device. The microfluidic platform previously developed [12] was modified (Figure S1) and coupled to real-time imaging using two different imaging techniques. Differential interference contrast (DIC) was used to detect cytoplasmic motion prior to cycle 10 in *wild-type* embryos, and a multi-point confocal system was used to detect nuclear motion in embryos expressing His2AvD-GFP (histone-eGFP) [14] (see Methods).

Experiments characterizing cytoplasmic motion with DIC microscopy were carried out using embryos exposed to the temperature step within 20 minutes post-fertilization. Embryos were placed in the device without removing the chorion, and two laminar streams of paraffin oil [15] were used to maintain each half of the embryo at a different temperature. Paraffin oil was used to facilitate imaging through the chorion using DIC (see Methods). Images of the mid-plane of the embryo were captured every 45 seconds and constructed into a time-series.

Experiments characterizing nuclear motion using confocal microscopy were carried out in embryos exposed to the temperature step between cycles 9 and 10 to ensure viability. Embryos that were exposed to the temperature step and imaged using confocal microscopy from fertilization displayed low viability, which could either be due to the embryos not being fertilized, or to the combined stresses of the temperature step and photodamage. Therefore, embryos were staged outside of the microfluidic device and embryos in cycle 8 were mounted in the device. Images were taken beginning at nuclear division cycle 11 and continuing in 45-second intervals for 30 to 40 min. Images taken to monitor development outside of this time window were captured only every three to five minutes to minimize photodamage. In all experiments, at each time point thirty to thirty-four frames were taken through the embryos from the surface to the mid-plane.

### Dramatic Motion of Cytoplasm and Nuclei is Observed in Embryos Exposed to a Temperature Step

During normal development under uniform environmental temperature, nuclei divide within the cytoplasm during nuclear division cycles 1–9. During this time, the embryo undergoes two critical processes: 1) axial expansion during cycles 4–6 and 2) cortical migration during cycles 8–9 [16]. During axial expansion, nuclei migrate from the anterior to become evenly dispersed throughout the cytoplasm. Axial expansion is accompanied by bidirectional flow of cytoplasm, or fountain streaming, as a result of the ordered nuclear and cytoskeletal rearrangement [17]. Apart from fountain streaming during cycles 4–6, movement of cytoplasm is minimal during cycles 1–9 [18], [19]. During cortical migration, nuclei migrate towards the surface of the embryo [15]. By cycle 10, nuclei reach the surface of the embryo and form an evenly dispersed monolayer, where they undergo an additional four divisions prior to cellularization [20]. During cycles 10–14, movement of nuclei is minimal at the surface of the embryo.

**Figure 1:**
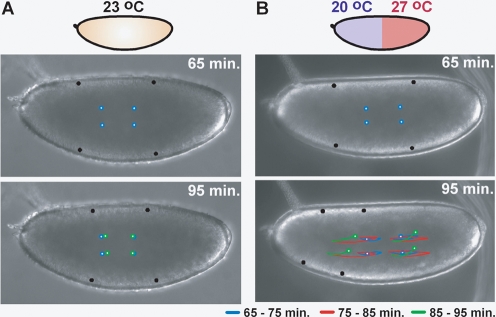
Dynamics of cytoplasmic movement in *wild-type* embryos placed in a microfluidic device under fluid flow of uniform temperature (A) and in a temperature step (B). Black particle tracers were added to the images by the image analysis software to facilitate visualization of the movement at the surface of the embryo. Similarly, blue, red, and green particle tracers were added to facilitate visualization of movement within the cytoplasm from 65–75 minutes, 75–85 minutes, and 85–95 minutes, respectively. (A) The embryo at uniform temperature of 23°C displayed minimal movement at the surface (black circles) and in the cytoplasm of the embryo (overlapping colored circles). (B) The embryo in a temperature step with anterior at 20°C and posterior at 27°C showed dramatic movement at the surface (black circles) and in the cytoplasm (colored circles). See Movie S1.

**Figure 2:**
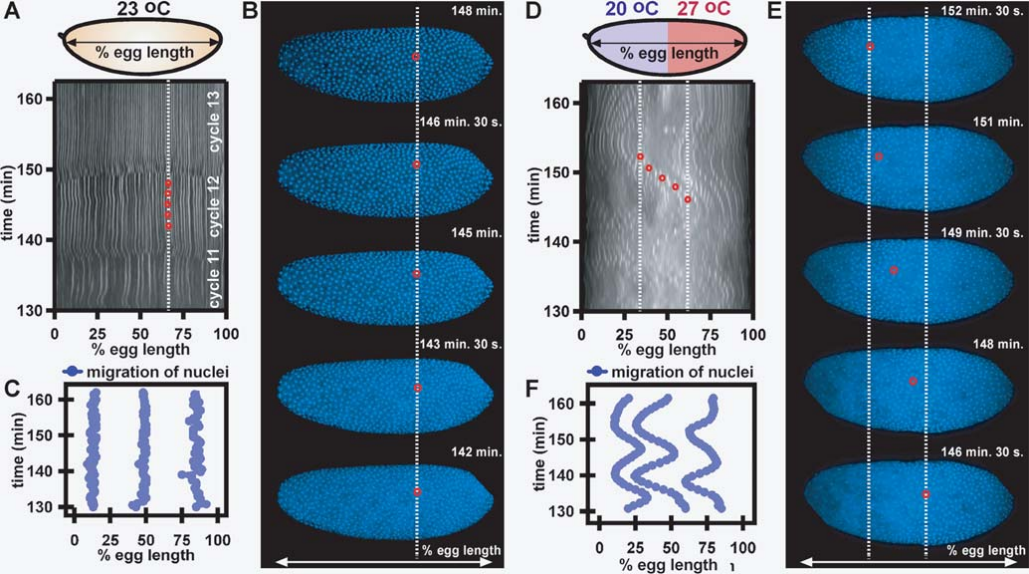
Dynamics of nuclear division cycles 11–13 in embryos expressing histone-eGFP developing in a microfluidic device under fluid flow of uniform temperature and in a temperature step. (A–C) Embryo at uniform 23°C. (A) A space-time plot of the profile of nuclei shows that nuclei remain in the same positions along the length of the embryo during cycles 11–13. White (high histone-eGFP intensity) correlates to the presence of a nucleus, and black (low histone-eGFP intensity) correlates to the absence of a nucleus. (B) Time-lapse images of the embryo in (A) from 142 to 148 min of development. The red circle tracks the position of one nucleus, and this position is also shown on the space-time plot in panel A. (C) A plot of the position of three nuclei over time shows that the positions of nuclei remain stable during cycles 11–13. See Movie S2. (D–F) Embryo with anterior at 20°C and posterior at 27°C. (D) A space-time plot of the profile of nuclei shows that nuclei move a maximum of 40% along the length of the embryo during cycles 11–13. Nuclei exhibited net movement from the warm half of the embryo to the cool half of the embryo. White correlates to the presence of a nucleus (high histone-eGFP intensity), and black correlates to the absence of a nucleus (low histone-eGFP intensity). (E) Time-lapse images of the embryo in (D) from 146 to 152 min of development. The red circle tracks the migration of one nucleus, and this position is also shown on the space-time plot in panel D. (F) A plot of the position of three nuclei over time shows that the position of the nuclei oscillates from posterior to anterior. See Movie S3.

Embryos exposed to uniform temperature in a microfluidic device display normal behavior during both earlier (cycles 1–9) (Figure 1A, Movie S1, top panel) and later (cycles 10–14) (Figure 2A–C, Movie S2) nuclear division cycles. Cytoplasmic motion was quantified in *wild-type* embryos by tracking particles prior to cycle 10 and nuclear motion was quantified by tracking nuclei at the surface of the embryo after cycle 10 (see Methods for a detailed explanation of data analysis). At uniform 23°C in a microfluidic device, cytoplasmic movement was minimal in *wild-type* embryos between 65 and 95 minutes of development (Figure 1A, Movie S1, top panel), as shown by particle tracers both at the surface (Figure 1A, black dots) and within the cytoplasm (Figure 1A, blue, red, and green dots). Nuclear motion was also minimal in a histone-eGFP [14] stock, used to facilitate visualization of nuclei during nuclear division cycles 10–14 (Figure 2A–C, Movie S2). Two methods were used to quantify motion of nuclei in embryos between nuclear division cycles 11–13. A linescan was taken from the anterior to the posterior pole of the embryo, and bulk nuclear motion was detected by measuring the fluorescence intensity profile of the histone-eGFP labeled nuclei along the line over time. A space-time plot was generated from this data showing nucleus position as a function of egg length over time (0% egg length corresponds to the anterior pole and 100% egg length corresponds to the posterior pole), where white indicates presence of a nucleus (high fluorescence intensity) and black indicates absence of a nucleus (low fluorescence intensity) (Figure 2A). Additionally, individual nuclei at different positions in the embryo (15, 50, and 85% egg length) were tracked as a function of egg length over time (Figure 2C). As seen in both analyses (Figure 2A,C), images taken from the time series (Figure 2B), and the movie (Movie S2) nuclei display minimal motion over time and during nuclear division cycles, moving on average much less than 10% egg length during nuclear division cycles 11–13.

In contrast to embryos developing at uniform temperature, embryos that developed in a temperature step with anterior at 20°C and posterior at 27°C displayed both severe cytoplasmic (Figure 1B, Movie S1, bottom panel) and nuclear motion (Figure 2D–F, Movie S3).

At earlier developmental stages prior to nuclear division cycle 10, dramatic oscillatory movement of cytoplasm was observed in *wild-type* embryos exposed to a temperature step (Figure 1B, Movie S1, bottom panel). It is critical to note that the oscillations took place after fountain streaming and were therefore not due to the natural migration of nuclei from the anterior to the posterior of the embryo. Furthermore, net movement was in the opposite direction from that caused by fountain streaming. The period of oscillations was on the order of 10 minutes, approximately the time of one nuclear division cycle, and the cytoplasm displayed a net movement from the warm half of the embryo to the cool half of the embryo. Given this dramatic cytoplasmic movement, formation of a stable Bcd gradient prior to corical migration would be difficult, as freely diffusing Bcd would presumably be mixed within the cytoplasm.

Likewise, after nuclear division cycle 10, dramatic motion of nuclei at the cortex was observed in histone-eGFP embryos exposed to a temperature step (Figure 2D–F, Movie S3). Nuclei displayed bulk oscillatory motion along the antero-posterior axis of the embryo, as seen in the space-time plot (Figure 2D), images from the time series (Figure 2E), and movie (Movie S3). Individual traced nuclei originating at 20, 65, and 85% egg length midway between the dorso-ventral axis moved up to 40% egg length during nuclear division cycles 11–13 (Figure 2F). Surprisingly, when this embryo was removed from the temperature step and imaged every 5 minutes to monitor development, the embryo recovered from the severely abnormal nuclear dynamics during cycles 11–13 and gastrulated (Figure S2). Therefore, the dramatic oscillatory motion observed during cycles 11–13 was not a result of photodamage or fluid shear, an observation which is in agreement with the results obtained with flow at uniform temperature (Figure 1A, and Figure 2A–C). Rather, the motion is presumably due to the more rapid nuclear divisions in the warmer half of the embryo causing an overcrowding of nuclei and an accompanying migration of nuclei from the warm posterior of the embryo to the cool anterior of the embryo. Nuclear motion is correlated with cell cycle, and the oscillatory behavior is presumably due to an accompanying opposite migration of nuclei towards the posterior of the embryo after nuclei in the cool anterior half undergo a division (Figure S3, Movie S3).

We emphasize that not all embryos displayed such dramatic nuclear movement during cycles 11–13. Three of four embryos displayed the type of motion presented in Figure 2D–F, and the fourth embryo displayed less severe motion. We hypothesize that the range of abnormal motion observed is due to either natural variation in the behavior of the embryos in the temperature step, slight differences in the nuclear division cycle at which embryos were staged (between cycles 8 and 9), or slight variations in the positions of the embryos in the channel, resulting in small variations of the flow field.

Prior to gastrulation, these embryos ultimately equalized nuclear density by undergoing a nuclear division in only the cool half of the embryo (Figure S4, Movie S4). After this division, the duration of the nuclear division cycle in the anterior, cool half of the embryo was on the order of 10 minutes, much shorter than expected for cycle 14 at 20°C.

### The Bicoid Gradient is Disrupted by Nuclear Motion and is Highly Abnormal as a Function of Egg Length

During normal development, the Bcd protein is localized within nuclei during interphase and leaves nuclei during mitosis. After each mitosis, the Bcd level within a given nucleus returns to nearly the same concentration, as measured originally [8] by fluorescence intensity of Bcd-eGFP. Overall, the Bcd gradient remains stable throughout cycles 10–14.

The dramatic motion of nuclei and its ultimate correction that we observed in embryos exposed to the temperature step raised two questions concerning the Bcd gradient. Given the oscillatory motion of nuclei, is the Bcd gradient, which is localized to nuclei during nuclear division cycles 10–14, also oscillatory? If the Bcd gradient is disrupted during nuclear division cycles 11–13, does the embryo correct the gradient, as it corrects for differences in nuclear density?

To determine the dynamics of the Bcd gradient in embryos exposed to uniform temperature of 23°C and to a temperature step with anterior at 20°C and posterior at 27°C in a microfluidic device, we observed Bcd-eGFP *in vivo* in real-time using the scanning-array confocal system described above. Like the experiments using histone-eGFP embryos, in order to ensure viability we staged the Bcd-eGFP embryos to cycle 8, exposed them to the temperature step beginning at cycles 9–10, and imaged them only from cycles 11–13 every 45 seconds.

The highly amplified EM CCD camera that we used to acquire images allowed for lower exposure times and further minimized photodamage, but this camera cannot make truly quantitative measurements. In addition, the scanning-array confocal system does not provide perfectly uniform spatial imaging. Nevertheless, we quantified the results by creating a calibration curve of standard solutions and correcting for the spatial non-uniformities (see Methods for details on image analysis). We tested the developed protocol by applying it to histone-eGFP images, in which each nucleus should be the same intensity. When corrected, the deviation in average intensity per nucleus was within 10%, suggesting that the protocol adequately corrects for non-uniformities.

**Figure 3:**
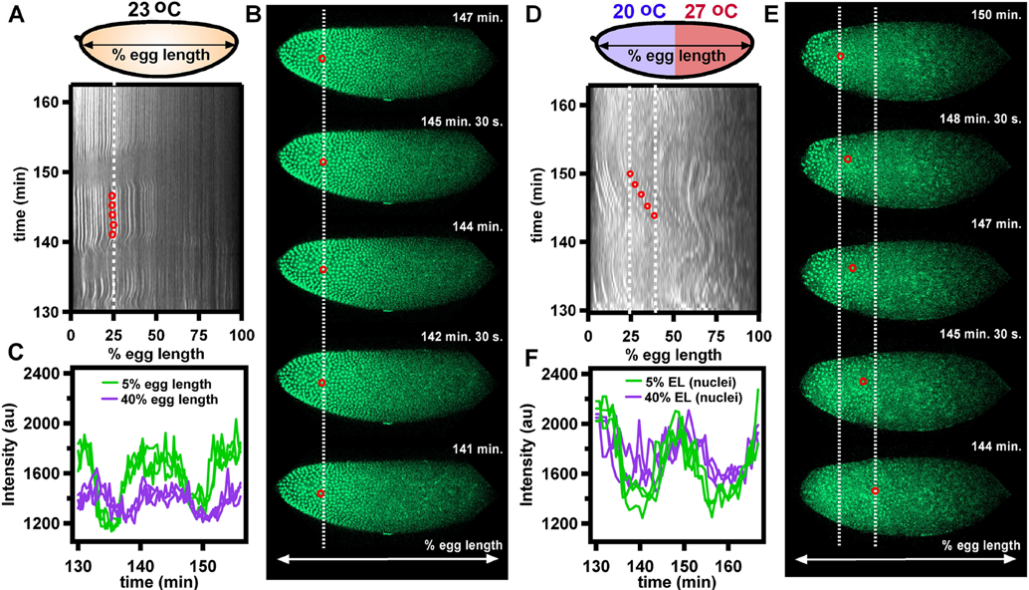
Dynamics of the Bcd gradient during cycles 11–13 in an embryo expressing Bcd-eGFP in a microfluidic device under fluid flow of uniform temperature and in a temperature step. (A–C) Embryo at uniform 23°C. (A) A space-time plot of the intensity of Bcd shows that the Bcd profile remains stable along the egg length of the embryo during cycles 11–13. White correlates to the presence of Bcd (high Bcd-eGFP intensity), and black correlates to the absence of Bcd (low Bcd-eGFP intensity). (B) Time-lapse images of the embryo in (A) from 141 to 157 min of development. The red circle tracks the location of one nucleus, and this position is also shown on the space-time plot in panel A. (C) A plot of the intensity of three nuclei at 5% egg length (green) and three nuclei at 40% egg length (purple), shows that the intensity of Bcd in the nuclei remains stable over division cycles 11–13. See Movie S5. (D–F) Embryo with anterior at 20°C and posterior at 27°C. (D) A space-time plot of the intensity of Bcd shows that the Bcd profile does not remain stable along the ventral length of the embryo during cycles 11–13. White correlates to the presence of Bcd (high Bcd-eGFP intensity), and black correlates to the absence of Bcd (low Bcd-eGFP intensity). (E) Time-lapse images of the embryo in (D) from 144 to 150 min of development. The red circle tracks the location of one nucleus, and this position is also shown on the space-time plot in panel D. (F) A plot of the intensity profile of three nuclei at 5% egg length (green) and three nuclei at 40% egg length (purple), shows that the Bcd intensity in a given nucleus remains constant over time. However, the intensity at 5% and 40% is nearly the same, showing the lack of a gradient over a significant portion of the embryo. See Movie S6.

Embryos exposed to a uniform temperature of 23°C in a microfluidic device displayed a normal Bcd profile that was precise over nuclear division cycles 11–13 (Figure 3A–C, Movie S5), as previously reported [8] for embryos developing under standard conditions outside of a microfluidic device. A linescan was taken from the anterior to the posterior pole of the embryo and bulk motion of Bcd was detected by measuring the fluorescence intensity profile of the Bcd-eGFP fusion protein along the line over time. A space-time plot was generated from this data showing Bcd as a function of egg length over time (0% egg length corresponds to the anterior pole and 100% egg length corresponds to the posterior pole), where white indicates presence of Bcd (high fluorescence intensity), and black indicates absence of Bcd (low fluorescence intensity) (Figure 3A). The maximum intensity of Bcd in a single nucleus during interphase was also stable over nuclear division cycles 11–13 (Figure 3C), as previously reported [8]. As expected, the embryo developing under uniform temperature had a lower intensity of Bcd at 40% egg length than at 5% egg length (p<.00000001). The agreement between the results from embryos developing in the microfluidic device and results previously reported [8] confirmed that our setup and imaging method did not adversely affect development.

In contrast, Bcd-eGFP embryos exposed to a temperature step with anterior at 20°C and posterior at 27°C had highly perturbed Bcd gradients that displayed oscillatory behavior similar to that of nuclei in histone-eGFP embryos exposed to the same temperature step. The embryo shown in Figure 3D–F and Movie S6 had nearly the same intensity of Bcd at both 40% and 5% egg length (p = .3533), showing that under the perturbed conditions of a temperature step, the Bcd gradient can be nearly abolished over half the body of the embryo. Interestingly, the area over which the gradient is abolished in this embryo covers several domains over which different concentrations of Bcd are required for expression of different zygotic genes, and the Bcd concentration at a given percent egg length is highly variable (Figure S5). Also, the maximum intensity of Bcd in a single nucleus during interphase was stable over nuclear division cycles 11–13, despite significant movement (Figure 3F and Figure S6B). The apparently stable Bcd intensity within a given nucleus could either be attributed to the lack of significant change in Bcd intensity over the majority of the anterior half of the embryo, or to Bcd being trapped within the energid (surrounding cytoplasm of a nucleus), even during a nuclear division cycle. A trapping mechanism was found to maintain the Dorsal protein gradient along the dorso-ventral axis during development of the embryo [21]. Cytoskeletal elements such as actin may play a similar role in trapping Bcd around nuclear regions (Figure S7A–C), and actin also has been shown to be disrupted in embryos exposed to a temperature step (Figure S7D–E).

Unlike correction of abnormal nuclear densities seen in histone-eGFP embryos, embryos with abnormal Bcd profiles did not always correct for abnormalities in the Bcd gradient by cycle 14. It is important to note that, like the histone-eGFP embryos, not all Bcd-eGFP embryos displayed the same response to the temperature step – one embryo showed severe distortions of the Bcd gradient, one (shown in Figure 3D–F) was slightly less severe, one had slight distortions, and one showed a normal Bcd pattern.

### Even-Skipped Patterning Remains Precise Despite an Abnormal Bicoid Gradient

Previous results [13] showed that Hunchback (Hb) and Even-skipped (Eve), the proteins of genes activated by the Bcd gradient, were patterned correctly in *wild-type* embryos that developed in a temperature step with anterior at 20°C and posterior at 27°C. It is surprising that such significant perturbations to the Bcd gradient could occur under conditions that give rise to normal patterning of both gap and pair-rule genes during cycle 14.

**Figure 4:**
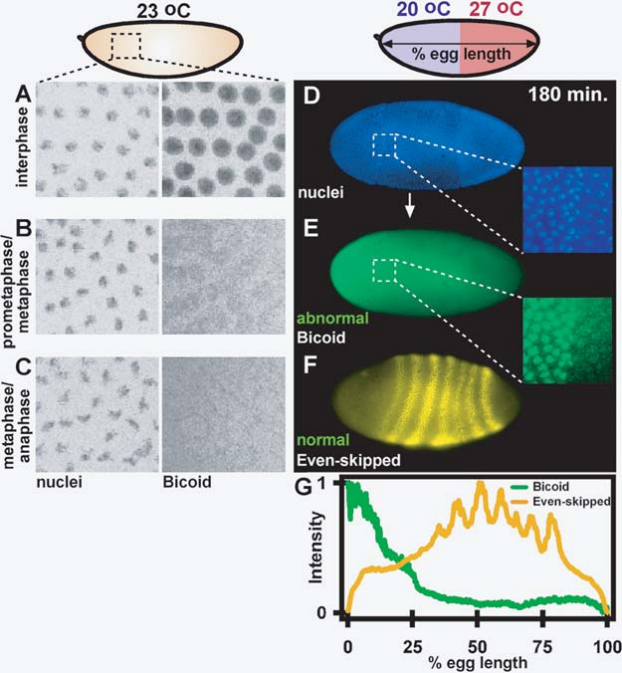
Bcd and Eve profile as determined by immunostaining of embryos in cycle 14 at uniform temperature and in a temperature step. (A–C) Embryos developed at uniform 23°C. Micrographs show a region of an embryo in interphase with Bcd localized to nuclei (A), in prometaphase/ metaphase with Bcd diffusely associated with nuclei (B), and in metaphase/anaphase with Bcd dissociated from nuclei and associated presumably with the cytoskeleton (C). (D–G) The embryo was exposed to a temperature step with anterior 20°C and posterior 27°C and then removed during cycle 14. Fluorescent micrographs show nuclei (D) and Bcd (E) in embryos developed in a temperature step. The Bcd gradient is abnormal with Bcd localized to nuclei only behind a region of abnormal nuclear morphology. (F) Eve stripes are expressed in the correct positions despite the abnormal Bcd intensity profile and abnormal morphology of nuclei in the cortex of the embryo. (G) Plot of Bcd and Eve normalized intensities as a function of percent egg length.

To verify that abnormal Bcd during cycles 11–13 in bcd-eGFP embryos can give rise to normal patterning during cycle 14 to gastrulation, we imaged a bcd-eGFP embryo using the same protocol as embryos shown in Figure 3, removed the embryo from the microfluidic device, and immunostained the embryo for Eve expression. While Bcd was disrupted during cycles 11–13 in this embryo, Eve patterning was normal (Figure S8). To further show that abnormal Bcd can lead to normal Eve patterning, we determined the intensity profile of Bcd and Eve in immunostained *wild-type* embryos that were placed in a microfluidic device exposed to a temperature step approximately 20 minutes after fertilization. Eve patterning was normal (Figure 4F,G) despite abnormal Bcd (Figure 4E,G) and abnormal morphology of the nuclei (Figure 4F) in the cortex of the embryo during cycle 14. Interestingly, Bcd in neighboring nuclei in the anterior half of the embryo (inset of Figure 4E) resembled the Bcd profile in embryos undergoing different phases of the cell cycle under uniform temperature (Figure 4A–C). Bcd expression was normal in control embryos that were developed on a molasses plate, fixed, and immunostained (Figure S9), confirming that the abnormalities seen in the Bcd expression in bcd-eGFP embryos exposed to the temperature step were not due to immunostaining protocols. Together, these experiments corroborate that a perturbed Bcd gradient during cycles 11–13 can lead to normal patterning during cycle 14 to gastrulation.

## Discussion

Temperature affects the rate of the majority of biochemical processes. Cells and organisms compensate for changes in temperature, and we are beginning to understand how this compensation could occur, especially in reference to circadian clocks [22], [23]. Changes in temperature have been shown to profoundly influence both embryonic processes, such as rate of nuclear divisions, and the shape of maternal gradients, such as that of Bcd [11]. These effects are presumably due to changes in the relative rates of transcription, translation, enzymatic modifications, and degradation. However, embryos of many classes of organisms develop normally within a large range of temperatures. Determining compensation mechanisms that embryos exploit in order to thrive in their ever changing internal and external environments could provide valuable insight into cellular robustness in general.

Under ideal, spatially uniform conditions, *Drosophila* embryos remarkably coordinate rapid nuclear divisions while converting the input of maternal factors such as Bcd into highly precise patterns of gene expression. Two questions have been the focus of research across disciplines: (1) How does the embryo coordinate the two distinct processes of nuclear divisions and protein patterning? and (2) How is robust patterning achieved? In answering the latter, work using immunostaining to detect Bcd implied that an imprecise Bcd gradient gives rise to precise gap and pair-rule gene expression [11], and that the noise in the Bcd gradient must be compensated for. However, recent work imaging Bcd *in vivo* showed that the Bcd gradient is in fact precise, and that the precision of downstream Hb expression mirrored that of the Bcd gradient [9]. This result raised the question of whether robustness against fluctuations in maternal gradients is necessary during early embryogenesis [8], [10], and whether stability of the Bcd gradient is essential for precise patterning. The results presented here indicate that during syncytial blastoderm stages, the stability of the Bcd gradient is nonessential to its function.

Under the perturbed, non-uniform environment of a temperature step, both Hb [13] and Eve patterning [13] (Figure 4F) are precise during cycle 14. However, the nuclear division cycles and Bcd gradient preceding precise patterning are drastically different in comparison to embryos developing under normal conditions. Dramatic cytoplasmic motion, followed by nuclear motion at the surface of the embryo, disrupts the Bcd gradient. Neither words nor images suffice to describe the fascinating dynamics observed under these conditions, and we encourage the reader to view the movies provided in the supporting information. These results suggest that precise patterning is governed by a mechanism distinct from reading the absolute concentration of the Bcd gradient as a function of egg length.

The observed dynamics of the Bcd gradient in embryos exposed to a temperature step suggest that formation and maintenance of the Bcd gradient is more complex than predicted by production-diffusion-degradation models, and that the read-out of the Bcd gradient is more complex than determining positional information as a function of egg length. Two pools of Bcd exist in the embryo: one in the bulk cytoplasm that is presumably affected by motion of cytoplasm before cycle 10, and one within the nuclei or energids which is presumably affected by motion of nuclei after cycle 10 in embryos exposed to the temperature step.

Before cycle 10, when the Bcd gradient is formed, is it difficult to reconcile a mechanism in which Bcd freely diffuses on the scale of egg length to form a gradient over the majority of the body of the embryo, given the severe cytoplasmic motion observed in embryos exposed to the temperature step. This motion would presumably disrupt the Bcd gradient formed by the pool of Bcd within the cytoplasm, if the gradient were formed by diffusion of Bcd through the bulk cytoplasm. However, it is important to note that this motion would presumably not disturb the Bcd gradient within the nuclei, formed by the pool of Bcd localized within nuclei or energids.

Before cycle 10, it is possible that oscillatory cytoplasmic motion, occurring at the low value of the Reynolds number inside the embryo [24], may be partially reversible (i.e. a particle could return to the point at which it originated at the end of each oscillation) when flow is both oscillatory and symmetric relative to the gradient (Figure 1B). However, embryos exposed to the temperature step within 20 minutes after fertilization have a much higher fluorescence background (Figure 4E) in comparison to embryos that developed at uniform temperature (Figure S9), suggesting that Bcd not trapped within nuclei or the energid was at least partially mixed, and that the mixing observed was not reversible. To more conclusively exclude this possibility, efficient mixing could be generated by breaking symmetry of flow [25]–[27] relative to the Bcd gradient, either by chemically disrupting the cytoskeletal network or by local asymmetric heating.

After cycle 10, when a large pool of Bcd is localized within nuclei, maintenance of the Bcd gradient by any long-range diffusive mechanism that would require transport of Bcd between nuclei or energids is difficult to reconcile with our results. Rather, our results support mechanisms in which Bcd is trapped within a given energid and only displays short-range diffusion. In embryos exposed to the temperature step, nuclei carry Bcd as they migrate, and while the Bcd gradient is highly perturbed as a function of egg length, the Bcd concentration within a nucleus remains constant through several nuclear division cycles. Trapping of Bcd by cytoskeletal structures in nucleus-associated cytoplasmic domains may account for maintenance of the constant concentration of Bcd, similarly to the trapping observed for the Dorsal protein [21]. While the nuclear and cytoplasmic flows observed are inconsistent with diffusion, they do not exclude diffusive movement of Bcd relative to the cytoplasm.

Given that embryos exposed to temperature steps retain precise Hb [13] and Eve [13] (Figure 4F) patterning in cycle 14 despite an abnormal Bcd gradient, what governs robustness? Formally, four mechanisms could reconcile the observation of an abnormal Bcd gradient as a function of egg length and the precise patterning later in the same embryos. Below we describe the mechanisms, available experimental evidence to support them, and future experiments required to test them.

The first mechanism encodes positional information in the reference frame of the number of nuclei rather than as a function of absolute egg length. This mechanism can be made robust to nuclear migration if the following conditions are met: i) movement of nuclei does not perturb their relative order, akin to beads moving on a string or on wires of an abacus. This is predominantly the type of movement observed in embryos exposed to the temperature step (Figure 2D–F). ii) During nuclear divisions, daughter nuclei maintain the same encoding of their parent nuclei, which could be facilitated by cytoskeletal trapping. iii) Ultimately, correct density and spacing of nuclei is achieved within the embryo, as was observed experimentally in embryos developed in a temperature step (Figure S4). This assumption is critical in transforming the positional information encoded in nuclei into spatial information. The molecular cues that orchestrate the observed correction of nuclear density (Figure S4) remain to be established. Encoding within the reference frame of nuclei, rather than as a function of absolute egg length, also facilitates patterning of embryos of different sizes by the same mechanism. An alternative encoding in the reference frame of cytoskeletal elements could be tested by imaging Bcd and cytoskeletal elements *in vivo* in real-time. This mechanism may be tested using embryos double-labeled for Bcd and histones to detect nuclei, or Bcd and cytoskeleton.

The second mechanism involves regulation of the boundaries of gap gene expression by gap gene cross-regulation [28], [29], which become less coupled to Bcd over time [30], as previously proposed. Previous work has shown that expression of segmentation genes is more precise than could be accounted for by the Bcd gradient alone [9], [31]. While Bcd clearly activates expression of several gap domains, it is possible that gap gene cross-regulation governs the exact boundary position and sharpness of gap domains. Determining the variability of gap gene expression during early cell cycles (cycles 10–13) would be highly informative in addressing whether gap gene expression is initially variable in response to the Bcd gradient. However, we believe that linking the observed gap gene dynamics to the abnormalities in the Bcd gradient can be done conclusively only by observing both gap gene expression and Bcd *in vivo* in real-time in the same embryo. Live imaging experiments that simultaneously monitor Bcd and gap gene products in embryos exposed to the temperature step would answer three important questions: when gap domains are established, if gap domains are refined through later cross-repression or come up in the proper position despite and abnormal Bcd profile, and if the variability of gap domains is increased in these embryos.

Third, positional information from the Bcd gradient could be decoded earlier in development, as previously proposed [32]. To maintain this decoding during the motion observed on cycles 11–13, this mechanism would have to be combined with either the first or the second mechanisms above. This mechanism still has to be reconciled with the severe cytoplasmic mixing observed throughout development; this mixing would presumably cause an abnormal Bcd gradient.

Finally, one could postulate that positional information is encoded spatially, as a function of egg length, but that this encoding is not affected by cytoplasmic movements, if for instance positional information is encoded in either the perivitelline space or the plasma membrane, and then transmitted to the nuclei via signaling, as proposed for the establishment of the Dorsal gradient [21]. Technologies for microinjections [33] into the perivitelline space, and experiments similar to the ones performed to analyze the formation of the Dorsal gradient [21] could become useful for testing this mechanism. We are not aware of data in direct support of this mechanism, but we cannot formally exclude it.

Using microfluidic devices to perturb the environment around a developing embryo provided a stunning view of the dynamics of embryonic development and the correction mechanisms employed under stressful conditions. These experiments provided a number of hypotheses on the coupling among the dynamics of the cytoskeleton, the nuclei, and the Bcd morphogen gradient. Testing these hypotheses would require a further integration of experimental tools with modeling and may help us better understand the robustness of embryonic development.

## Materials and Methods

### Imaging embryos in a microfluidic device using DIC and Confocal Microscopy

Embryos imaged by using differential interference contrast (DIC) optics were mounted as previously described [12] and imaged using a Leica DM IRB inverted microscope and an ×20 0.4 NA objective.

When imaging embryos using confocal microscopy, the microfluidic device previously described [12] was coupled to a multi-point confocal system. To reduce photodamage and increase acquisition rate during real-time imaging, a Visitech Infinity 2-D array scanner confocal system with an array of 50 µm pinholes was mounted to a Leica DMI6000 inverted microscope. Bcd and histone eGFP-fusion proteins were excited *in vivo* by using a 491 nm diode laser. Images were acquired using a back-thinned electron multiplier CCD camera (16 bit, 512×512 pixels, Hamamatsu Photonics) and ×20 0.7 NA objective.

Modifications were made to the microfluidic device previously described [12] to accommodate confocal microscopy and higher numerical aperature objectives. The bottom half of the device was fabricated to be ∼500 µm for use with shorter working distance, higher numerical aperature objectives. The top half of the device was fabricated by curing a suspension of charcoal in the polydimethylsiloxane (PDMS) to reduce light scattering and increase sensitivity of fluorescence detection within the device.

### Staging Embryos and Mounting in Microfluidic Device

Embryos were collected over a 30 minute period, allowed to develop for 1 hour at 23°C, dechorionated, placed on a glass bottom petri dish, covered in 1× PBS buffer, and staged using DIC optics. Embryos in cycle 8–9 were removed from the glass-bottom petri dish and mounted in the microfluidic device, as previously described [12], [13]. The microfluidic device was clamped to a stage insert. For an illustration of the device setup, refer to Figure S1.

### Detecting Cytoplasmic Motion

*wild-type* embryos were mounted on Scotch #667 double-stick tape in a microfluidic device as previously described. The chorion was left on embryos mounted in the microfluidic device. To visualize cytoplasmic motion through the chorion, paraffin oil [15] was flowed over the embryos. A time-series of images were taken using a SPOT camera on a Leica DM IRB inverted microscope with differential interference contrast microscopy and an ×20 0.4NA objective. Images of the midplane were taken every 45 seconds.

### Detecting Nuclear Motion

His2AvD-GFP embryos [14] were mounted by the same procedure used to detect cytoplasmic motion and as previously described [12], [13]. The chorion was removed to facilitate detection of fluorescence. 1× PBS buffer was flowed over the dechorionated embryos. The scanning-array confocal system enabled real-time imaging of an entire plane of the embryo simultaneously. The rapid acquisition allowed by this system enabled the capture of stacks of images in the Z direction at each time point and the minimization of photodamage to the embryo. A time-series of images were taken using a highly amplified Hamamatsu EM CCD camera attached to the confocal system. This setup allowed detection of low intensity fluorescence. The EM CCD camera greatly amplified the low intensity signal, but the camera was not suitable for making truly quantitative measurements of concentration. However, we attempted to quantify the results by reporting values of intensities of background corrected images.

### Fluorescent Immunostaining

*wild-type* embryos were fixed in 3% formaldehyde in PEM buffer and immunostained using standard methods with anti-Bcd (mouse monoclonal) and anti-Even-skipped (rabbit polyclonal) primary antibodies and goat anti-mouse IgG (H+L) AlexaFluor 488 and goat anti-rabbit IgG (H+L) AlexaFluor 594 conjugated secondary antibodies (Molecular Probes). Bcd-eGFP embryos were fixed in 3% formaldehyde in PEM buffer and immunostained using standard methods with rabbit IgG AlexaFluor 647 conjugated anti-GFP (Molecular Probes). Actin was stained using AlexaFluor 594 phalloidin (Molecular Probes).

### Image Analysis and Image Processing

Images were acquired by using Simple PCI (Compix) and analyzed by using MetaMorph Imaging System (Universal Imaging Corp.). To correct for the gaussian distribution of illumination through the multi-point scanning array, images were taken 500 µm above the #1 cover-glass bottom of a petri dish filled with known concentrations of fluorescein with similar (plus or minus 100 au) maximum intensity to *Drosophila* images. *Drosophila* images were first divided by the fluorescein image with corresponding maximum intensity and then multiplied by the maximum intensity of the fluorescein image. Average intensity was recorded along a line (scanwidth of 10 pixels) along the antero-posterior axis of the embryo, mid-way between the dorso-ventral axis. The line was then converted into regions with a width of 5 pixels and a height of 10 pixels, and maximum and integrated intensities were measured in each region (Figure S10). Space-time plots and three-dimensional representations of data in the space-time plots were generated using the average intensity of Bcd. Maximum intensity within a region around a given nucleus was measured to track maximum Bcd intensity within individual nuclei over time. Fluorescent images were rotated, cropped, and false-colored using Photoshop for presentation in figures.

Particles in embryos imaged using DIC optics were manually traced and marked using MetaMorph Imaging System (Universal Imaging Corp.). Movement of nuclei was traced and quantified in histone-eGFP stocks imaged in real-time by using a sobel filter in MetaMorph Imaging System to outline each nucleus in white and contrast nuclei from the black, lower-intensity background (Figure S11).

Images of anti-GFP and phalloidin labeled embryos were acquired by using Leica software and processed by using Adobe Photoshop 6.0. Levels of images were adjusted to enhance contrast; all images were adjusted in the same manner.

## Supporting Information

Figure S1 Schematic of a microfluidic device coupled to confocal microscopy. The microfluidic device is clamped to a plate, which inserts into the motorized stage of the microscope, minimizing movement of the device relative to the microscope. A thin (∼500 µm) device is fabricated to accommodate a higher numerical aperature objective (×20, 0.7 N.A.).
(9.19 MB TIF)

Figure S2 The embryo shown in Figure 2D–E that was imaged from cycles 11–13 in a temperature step with anterior at 20°C and posterior at 27°C and then monitored at uniform 23°C at 180 minutes of development gastrulated and recovered from the temperature step. Images shown are from 180, 210, and 240 minutes of development.
(3.90 MB TIF)

Figure S3 Overlay of cell cycle phase as a function of egg length and nuclear motion quantified in Figure 2D of the histone-eGFP embryo from Figure 2D–F and Movie S2. The embryo was exposed to a temperature step with anterior at 20°C and posterior at 27°C. White squares correspond to a region of nuclei in metaphase, light blue squares correspond to a region of nuclei in telophase, and light brown squares correspond to a region of nuclei that is mixed metaphase and telophase. Nuclei move from the warm posterior half towards the cool anterior half after the embryo after the posterior half undergoes a nuclear division, presumably due to overcrowding of nuclei. The oscillatory behavior of nuclear movement is presumably due to a later division in the cool anterior half of the embryo which causes an opposite movement of nuclei back towards the posterior.
(3.60 MB TIF)

Figure S4 An embryo in a temperature step with anterior at 20°C and posterior at 27°C corrected for nuclear density by dividing only in the anterior half of the embryo. A) Space-time plot showing nuclear position over time (white corresponds to high fluorescence intensity or presence of a nucleus, and black corresponds to low fluorescence intensity or absence of a nucleus). Nuclei in the anterior half of the embryo divided at ∼200 minutes, approximately 10 minutes before the onset of gastrulation. B) Corresponding images at 195, 201, and 221 minutes from the time series.
(5.27 MB TIF)

Figure S5 Collapsed view of the space-time plots presented in Figure 3A and D shows the variability in the concentration of Bcd at a given point along the length of the egg over time. Each line is one time point from the time-series. All time-points between cycles 11–13 that were observed in real-time are shown. A) The concentration of Bcd at the mid-point of the embryo does not vary significantly over time in an embryo developed at uniform 23°C. B) The embryo exposed to a temperature step with anterior at 20°C and posterior at 27°C has a highly variable concentration of Bcd at the mid-point of the embryo over time. Given this result, it is difficult to reconcile a mechanism in which the embryo reads a given Bcd concentration as a function of egg length over time to activate precise zygotic gene expression. Surprisingly, both Hb and Eve patterning under both uniform temperature and temperature step conditions has been shown to be highly precise.
(3.10 MB TIF)

Figure S6 Bcd intensity within nuclei originating at 40% egg length at 130 minutes of development remains the same in both embryos developing at uniform 23°C and in a temperature step with anterior at 20°C and posterior at 27°C, despite drastic difference in the amount of nuclear motion. A) Nuclear motion of three nuclei in the embryo shown in Figure 3A–C, which developed at uniform temperature, was on the order of 2% egg length. B) Nuclear motion of three nuclei in the embryo shown in Figure 3D–F, which developed in a temperature step, was on the order of 20% egg length.
(7.47 MB TIF)

Figure S7 Bicoid protein is presumably trapped within the energid around a given nucleus, potentially by cytoskeletal elements such as actin. (A–C) Nuclei, actin, and Bicoid profiles from the anterior and posterior halves of an embryo developed at uniform 23°C. (A) Regions from the anterior and posterior halves of the embryo with nuclei in metaphase/anaphase. (B) Actin in these regions forms hexagonal rings around individual nuclei. (C) As the nuclei divide the Bicoid protein, localized in the head (appearing in only the left panel), appears diffuse and partially overlapping actin. (D–E) The actin network is disrupted at the boundary between high and low density nuclei in embryos exposed to a temperature step. (D) Nuclei detected by DAPI staining. A boundary is observed between high and low densities of nuclei. (E) Actin detected by phalloidin. The actin network is disrupted at the boundary between high and low density nuclei and appears to be highly compressed in the region of high density of nuclei.
(2.20 MB TIF)

Figure S8 An embryo exposed to a temperature step with anterior at 20°C and posterior at 27°C and imaged in real-time displays normal Even-skipped patterning during cycle 14 to gastrulation, as detected by removing the embryo from the microfluidic device at cycle 14 and immunostaining. Interestingly, Bcd remains abnormal in this embryo, despite precise Eve patterning.
(2.46 MB DOC)

Figure S9 Control embryos developed at 23°C and fluorescently immunostained for Bcd displayed a normal Bcd profile.
(1.66 MB TIF)

Figure S10 Comparison of Bcd intensity in the embryos shown in Figure 3, measured as an average intensity, integrated intensity or maximum intensity for one time point of the time-series.
(14.18 MB TIF)

Figure S11 Sobel filter used to track nuclei over time in embryos exposed to a temperature step.
(11.16 MB TIF)

Movie S1 Movies show the dynamics of cytoplasmic movement in developing embryos. To facilitate visualization of the movement at the surface of the embryo, black particle tracers were added to the images by using the image analysis software. (Top) In embryos developed in a microfluidic device at uniform 23°C, minimal cytoplasmic movement is observed. (Bottom) In embryos developed in a microfluidic device exposed to a temperature step with anterior (left) at 20°C and posterior (right) at 27°C, dramatic cytoplasmic movement is observed, with net movement from the warm to the cool half of the embryo.
(1.64 MB AVI)

Movie S2 A movie shows the dynamics of nuclear movement in a developing embryo in a microfluidic device at uniform temperature of 23°C. The nuclei were visualized by using the expression of histone-eGFP (blue).
(1.00 MB AVI)

Movie S3 A movie shows the dynamics of nuclear movement in a developing embryo in a microfluidic device exposed to a temperature step with anterior (left) at 20°C and posterior (right) at 27°C. The nuclei were visualized by the expression of histone-eGFP (blue).
(1.02 MB AVI)

Movie S4 A movie shows a partial nuclear division cycle only in the anterior (left, cool) half of an embryo developing in a microfluidic device exposed to a temperature step with the anterior (left) at 20°C and posterior (right) at 27°C. The nuclei were visualized by using the expression of histone-eGFP (blue).
(1.99 MB AVI)

Movie S5 A movie shows the dynamics of the Bcd gradient in a developing embryo in a microfluidic device at uniform temperature of 23°C. Embryos were expressing Bcd-eGFP (green).
(2.07 MB AVI)

Movie S6 A movie shows the dynamics of the Bcd gradient in a developing embryo in a microfluidic device exposed to a temperature step with anterior (left) at 20°C and posterior (right) at 27°C. Embryos were expressing Bcd-eGFP (green).
(0.73 MB AVI)
