# Supplementary figures and images for: A Precise Bicoid Gradient Is Nonessential during Cycles 11–13 for Precise Patterning in the Drosophila Blastoderm
Source: PLoS One. 2008 Nov 7;3(11):e3651. doi: 10.1371/journal.pone.0003651 (PMC2578877; doi:10.1371/journal.pone.0003651)

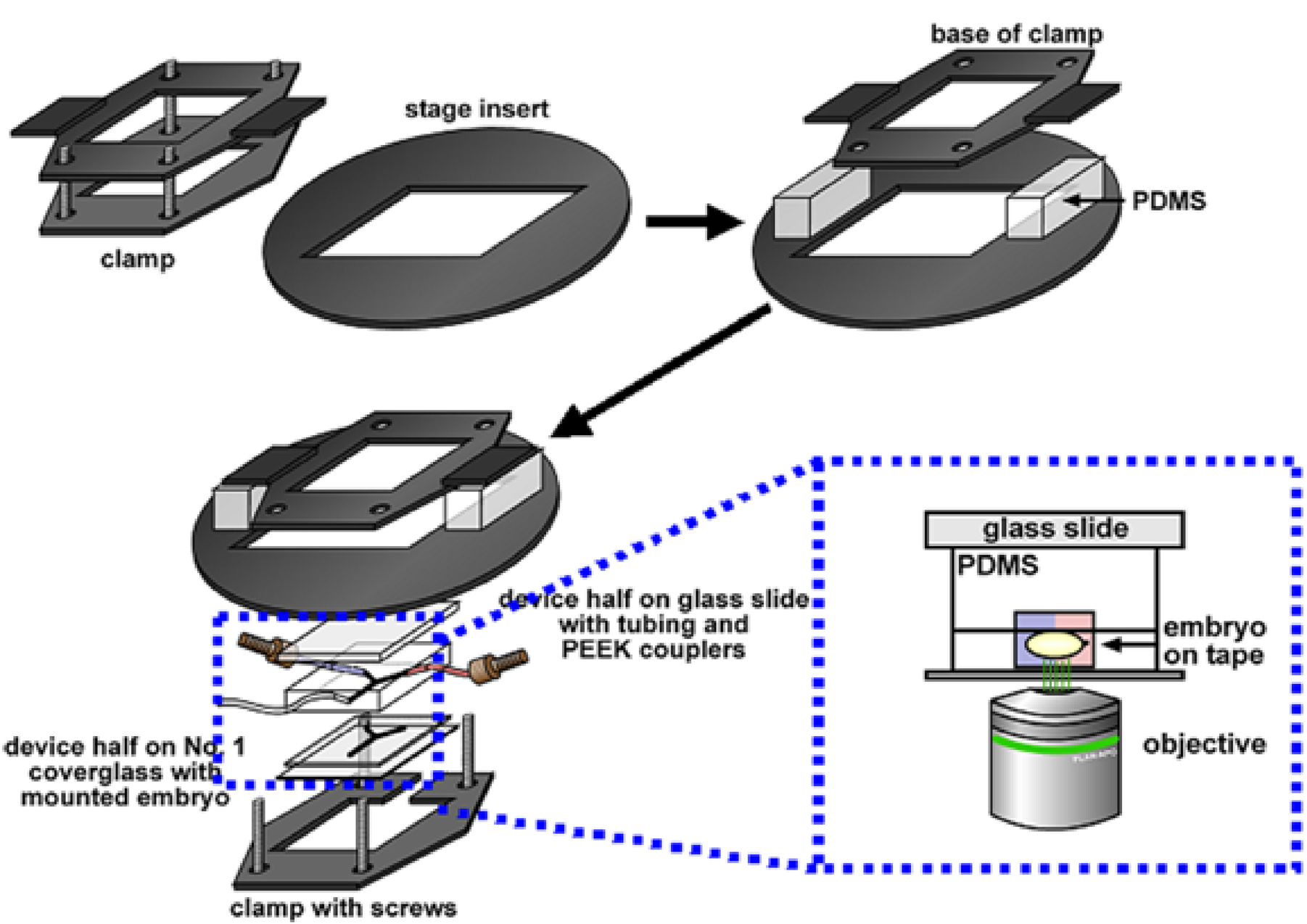

Supplement: Figure S1 — Schematic of a microfluidic device coupled to confocal microscopy. The microfluidic device is clamped to a plate, which inserts into the motorized stage of the microscope, minimizing movement of the device relative to the microscope. A thin (∼500 µm) device is fabricated to accommodate a higher numerical aperature objective (×20, 0.7 N.A.). (9.19 MB TIF) [file pone.0003651.s001.tif]

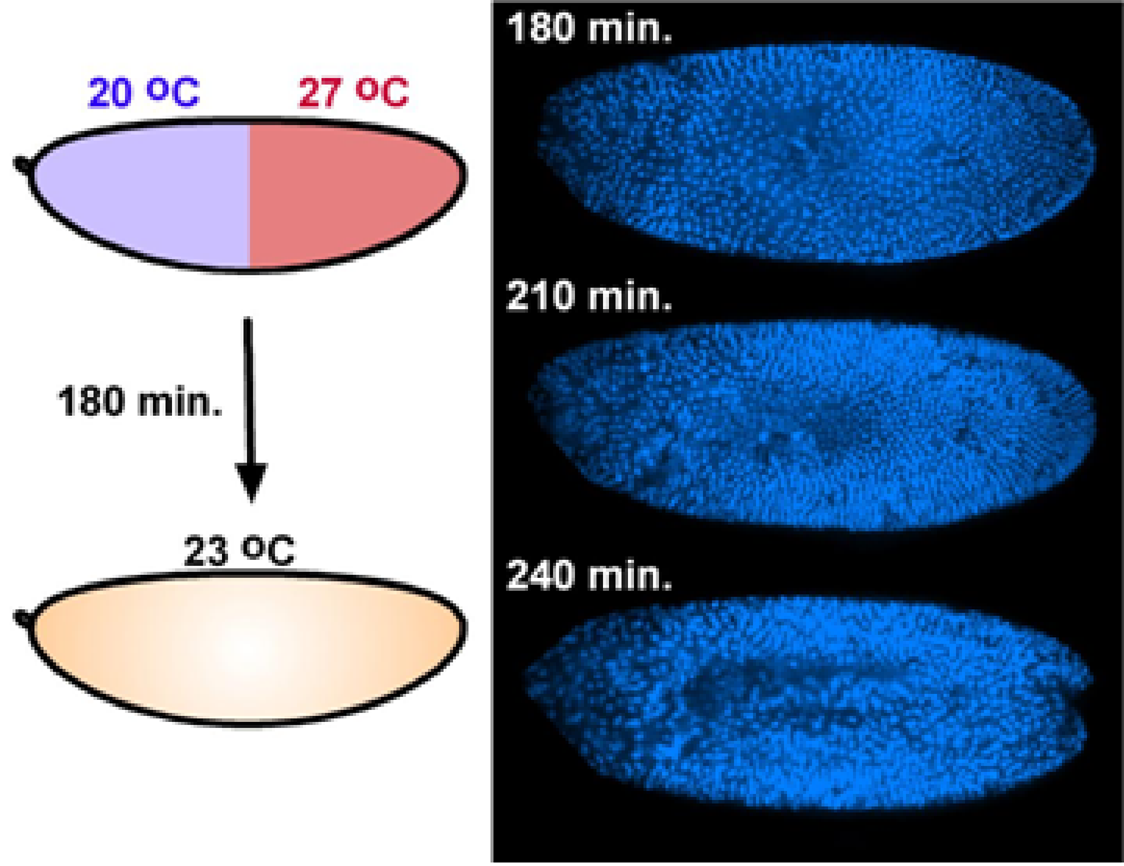

Supplement: Figure S2 — The embryo shown in Figure 2D–E that was imaged from cycles 11–13 in a temperature step with anterior at 20°C and posterior at 27°C and then monitored at uniform 23°C at 180 minutes of development gastrulated and recovered from the temperature step. Images shown are from 180, 210, and 240 minutes of development. (3.90 MB TIF) [file pone.0003651.s002.tif]

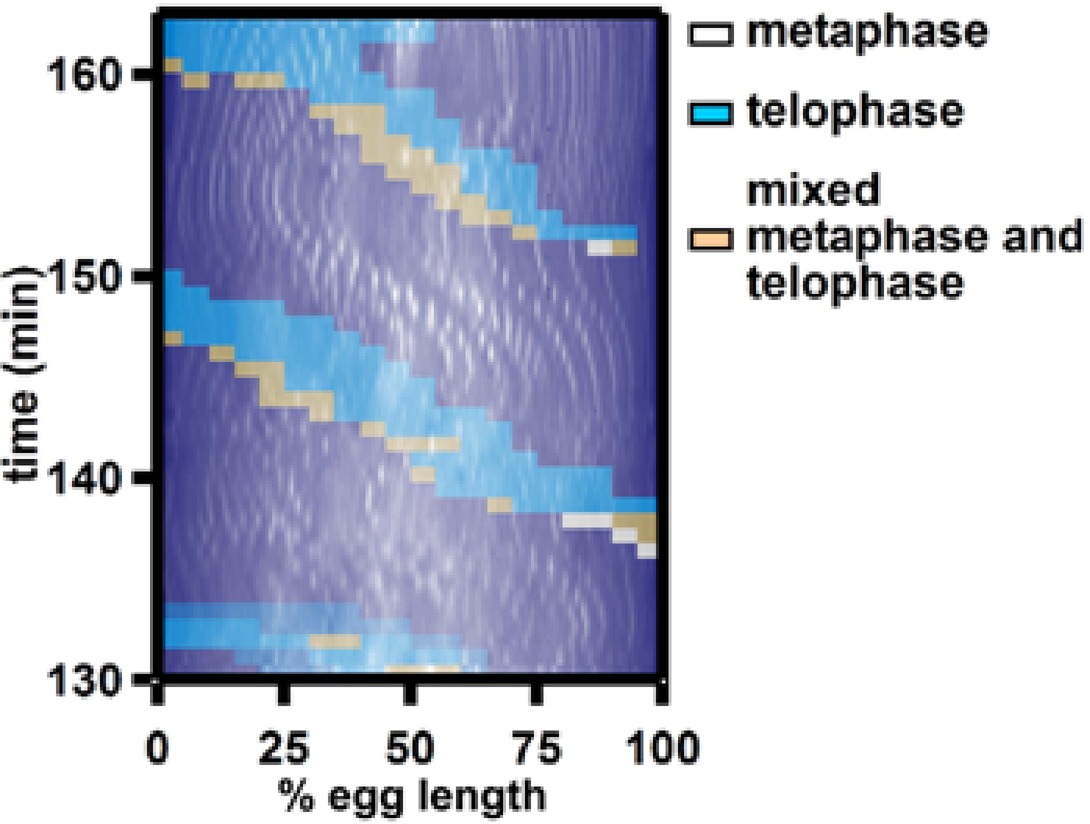

Supplement: Figure S3 — Overlay of cell cycle phase as a function of egg length and nuclear motion quantified in Figure 2D of the histone-eGFP embryo from Figure 2D–F and Movie S2. The embryo was exposed to a temperature step with anterior at 20°C and posterior at 27°C. White squares correspond to a region of nuclei in metaphase, light blue squares correspond to a region of nuclei in telophase, and light brown squares correspond to a region of nuclei that is mixed metaphase and telophase. Nuclei move from the warm posterior half towards the cool anterior half after the embryo after the posterior half undergoes a nuclear division, presumably due to overcrowding of nuclei. The oscillatory behavior of nuclear movement is presumably due to a later division in the cool anterior half of the embryo which causes an opposite movement of nuclei back towards the posterior. (3.60 MB TIF) [file pone.0003651.s003.tif]

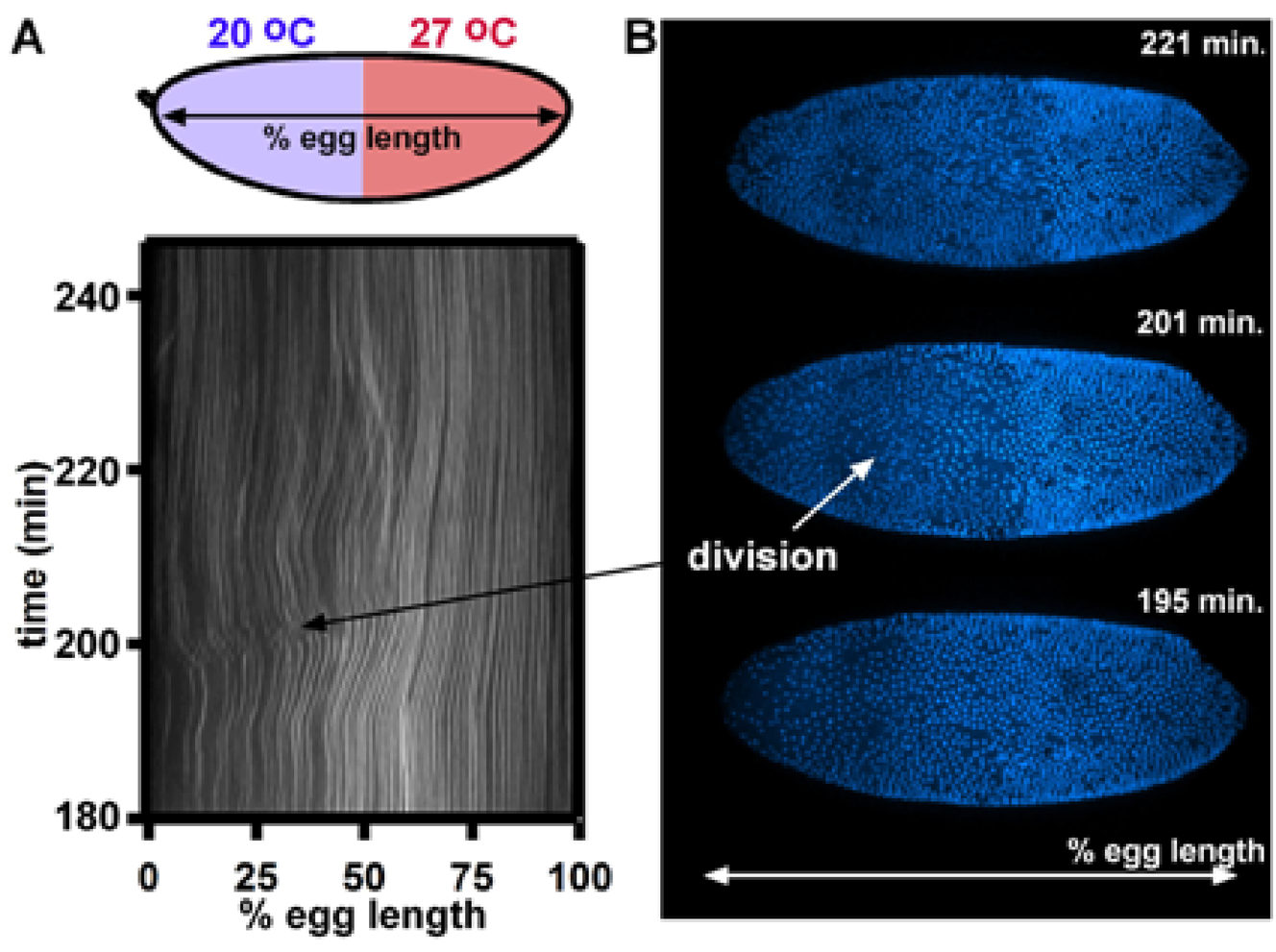

Supplement: Figure S4 — An embryo in a temperature step with anterior at 20°C and posterior at 27°C corrected for nuclear density by dividing only in the anterior half of the embryo. A) Space-time plot showing nuclear position over time (white corresponds to high fluorescence intensity or presence of a nucleus, and black corresponds to low fluorescence intensity or absence of a nucleus). Nuclei in the anterior half of the embryo divided at ∼200 minutes, approximately 10 minutes before the onset of gastrulation. B) Corresponding images at 195, 201, and 221 minutes from the time series. (5.27 MB TIF) [file pone.0003651.s004.tif]

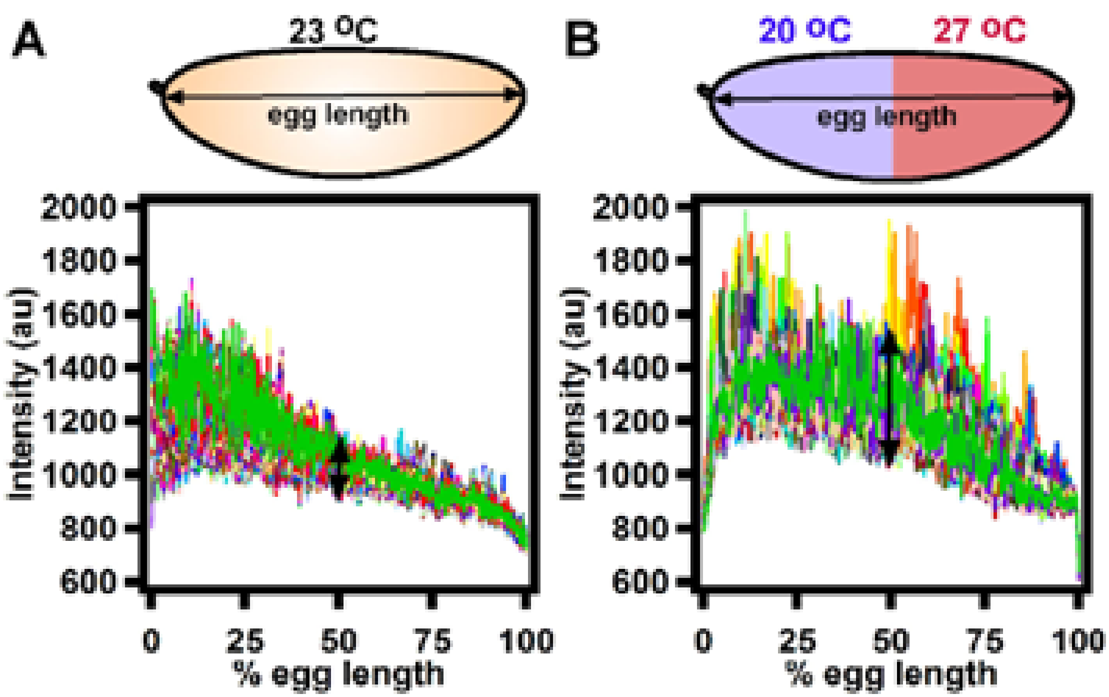

Supplement: Figure S5 — Collapsed view of the space-time plots presented in Figure 3A and D shows the variability in the concentration of Bcd at a given point along the length of the egg over time. Each line is one time point from the time-series. All time-points between cycles 11–13 that were observed in real-time are shown. A) The concentration of Bcd at the mid-point of the embryo does not vary significantly over time in an embryo developed at uniform 23°C. B) The embryo exposed to a temperature step with anterior at 20°C and posterior at 27°C has a highly variable concentration of Bcd at the mid-point of the embryo over time. Given this result, it is difficult to reconcile a mechanism in which the embryo reads a given Bcd concentration as a function of egg length over time to activate precise zygotic gene expression. Surprisingly, both Hb and Eve patterning under both uniform temperature and temperature step conditions has been shown to be highly precise. (3.10 MB TIF) [file pone.0003651.s005.tif]

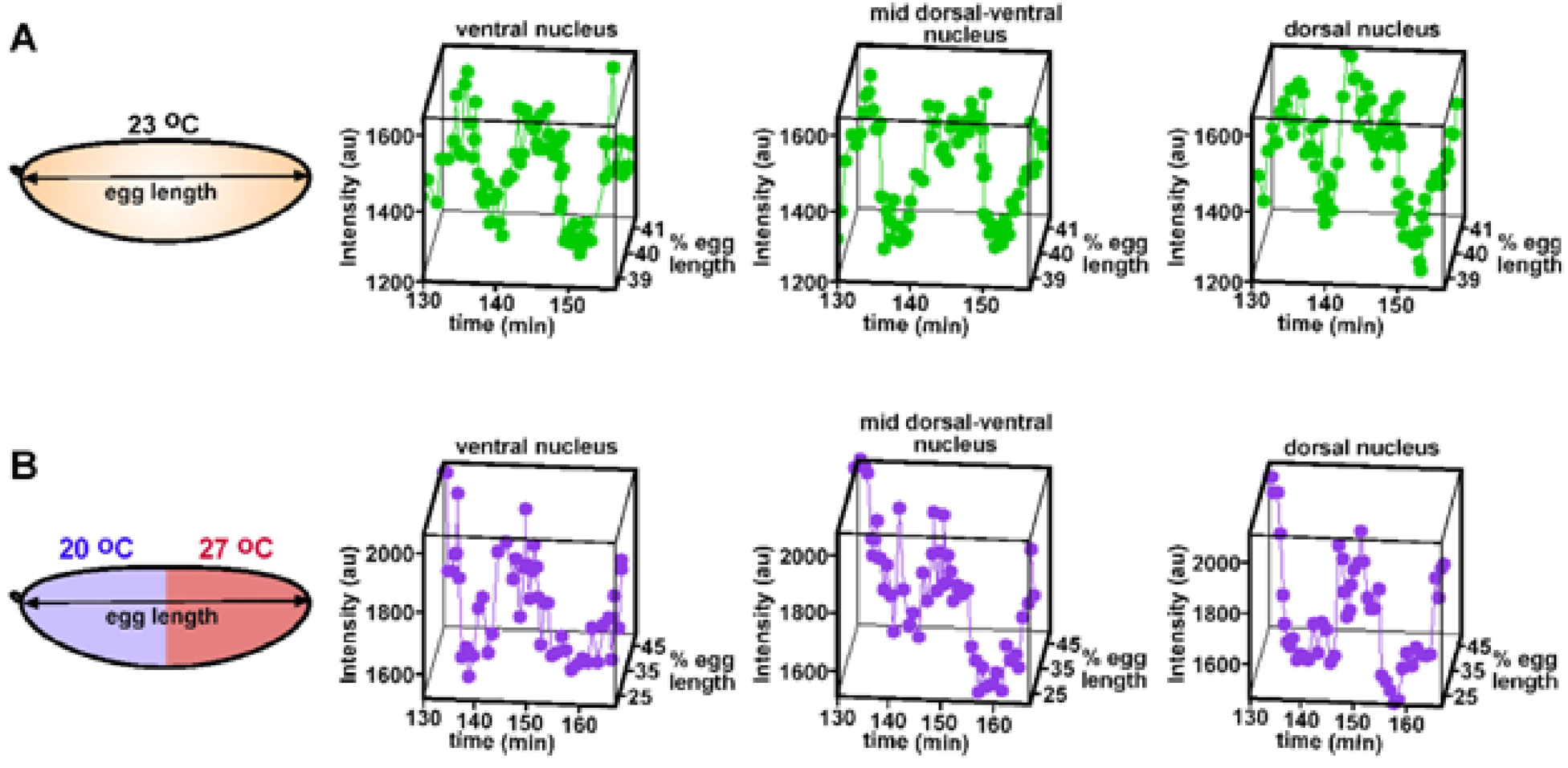

Supplement: Figure S6 — Bcd intensity within nuclei originating at 40% egg length at 130 minutes of development remains the same in both embryos developing at uniform 23°C and in a temperature step with anterior at 20°C and posterior at 27°C, despite drastic difference in the amount of nuclear motion. A) Nuclear motion of three nuclei in the embryo shown in Figure 3A–C, which developed at uniform temperature, was on the order of 2% egg length. B) Nuclear motion of three nuclei in the embryo shown in Figure 3D–F, which developed in a temperature step, was on the order of 20% egg length. (7.47 MB TIF) [file pone.0003651.s006.tif]

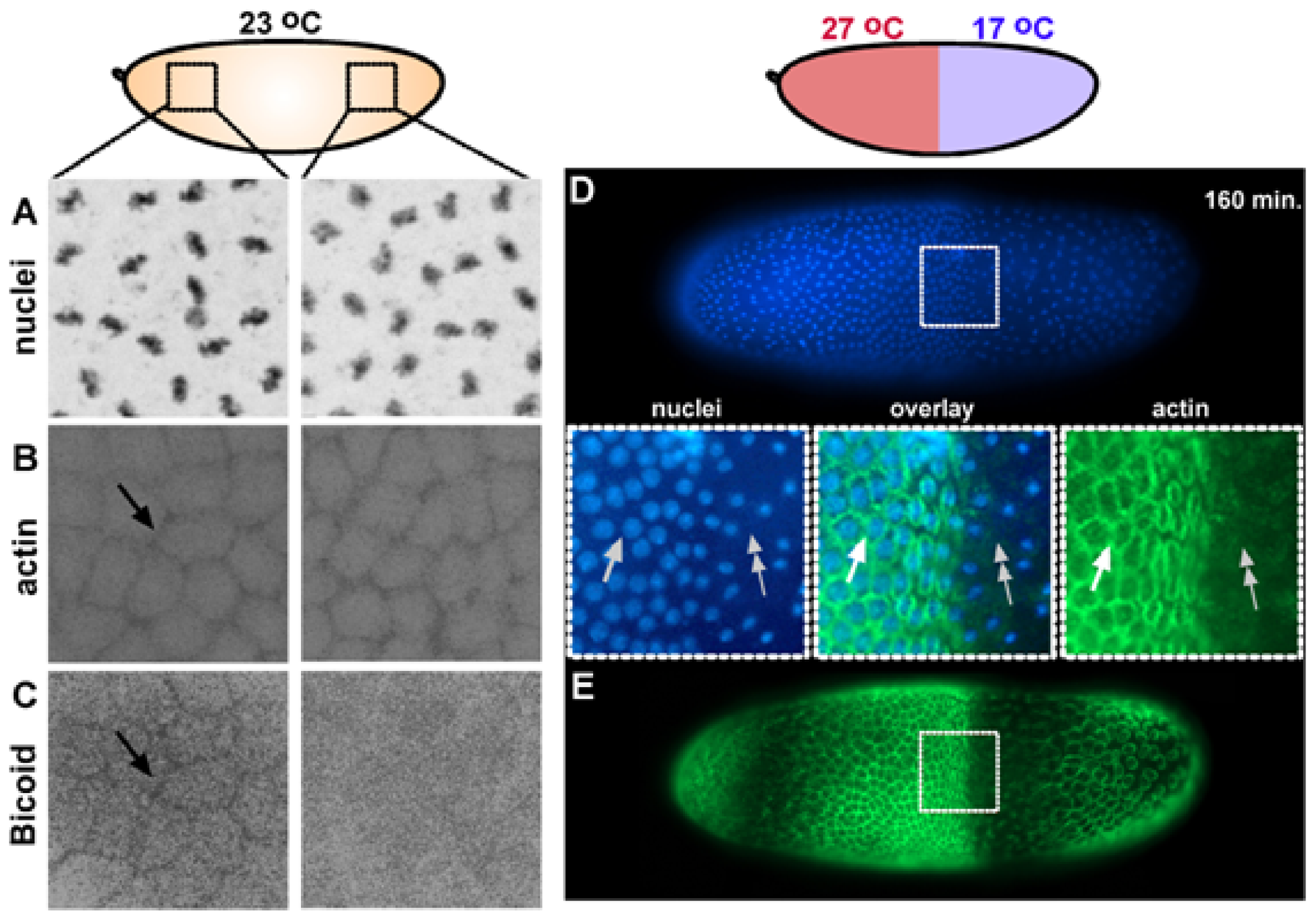

Supplement: Figure S7 — Bicoid protein is presumably trapped within the energid around a given nucleus, potentially by cytoskeletal elements such as actin. (A–C) Nuclei, actin, and Bicoid profiles from the anterior and posterior halves of an embryo developed at uniform 23°C. (A) Regions from the anterior and posterior halves of the embryo with nuclei in metaphase/anaphase. (B) Actin in these regions forms hexagonal rings around individual nuclei. (C) As the nuclei divide the Bicoid protein, localized in the head (appearing in only the left panel), appears diffuse and partially overlapping actin. (D–E) The actin network is disrupted at the boundary between high and low density nuclei in embryos exposed to a temperature step. (D) Nuclei detected by DAPI staining. A boundary is observed between high and low densities of nuclei. (E) Actin detected by phalloidin. The actin network is disrupted at the boundary between high and low density nuclei and appears to be highly compressed in the region of high density of nuclei. (2.20 MB TIF) [file pone.0003651.s007.tif]

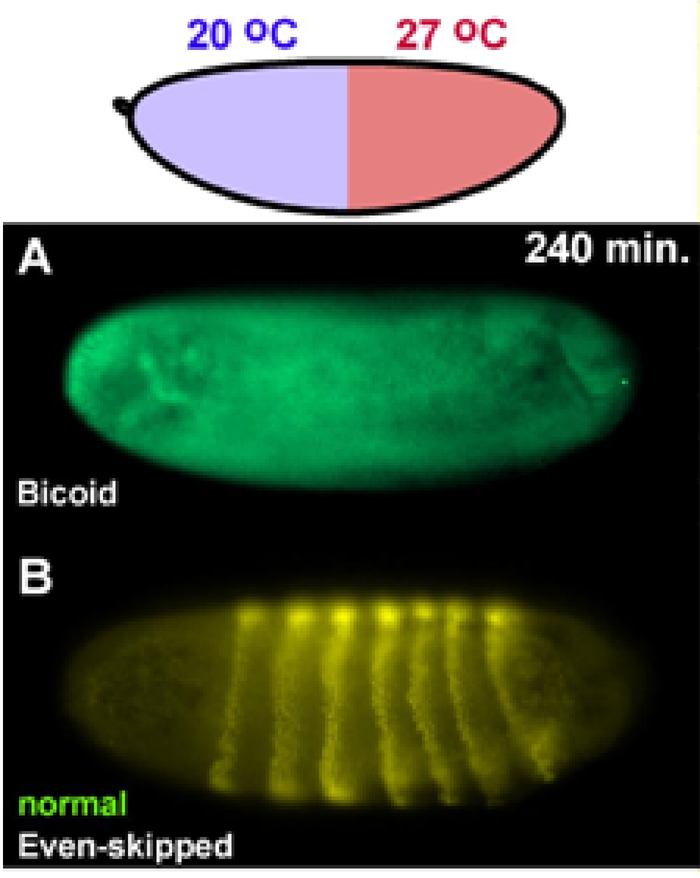

Supplement: Figure S8 — An embryo exposed to a temperature step with anterior at 20°C and posterior at 27°C and imaged in real-time displays normal Even-skipped patterning during cycle 14 to gastrulation, as detected by removing the embryo from the microfluidic device at cycle 14 and immunostaining. Interestingly, Bcd remains abnormal in this embryo, despite precise Eve patterning. (2.46 MB DOC) [file pone.0003651.s008.doc]

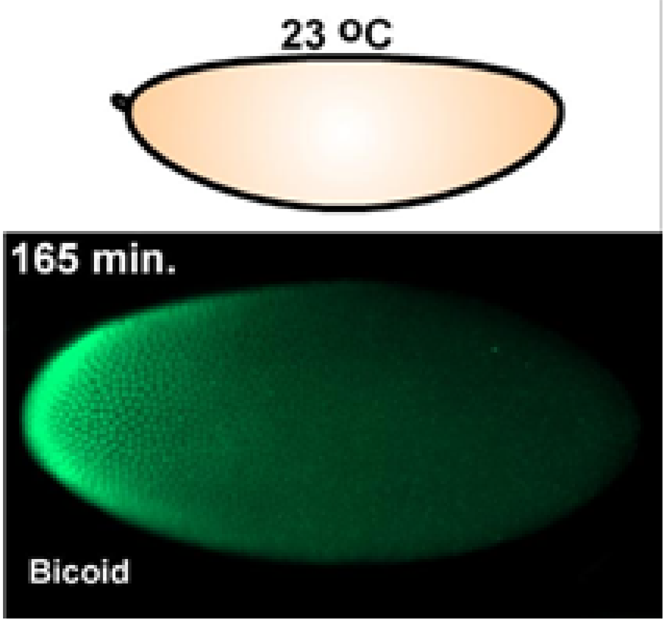

Supplement: Figure S9 — Control embryos developed at 23°C and fluorescently immunostained for Bcd displayed a normal Bcd profile. (1.66 MB TIF) [file pone.0003651.s009.tif]

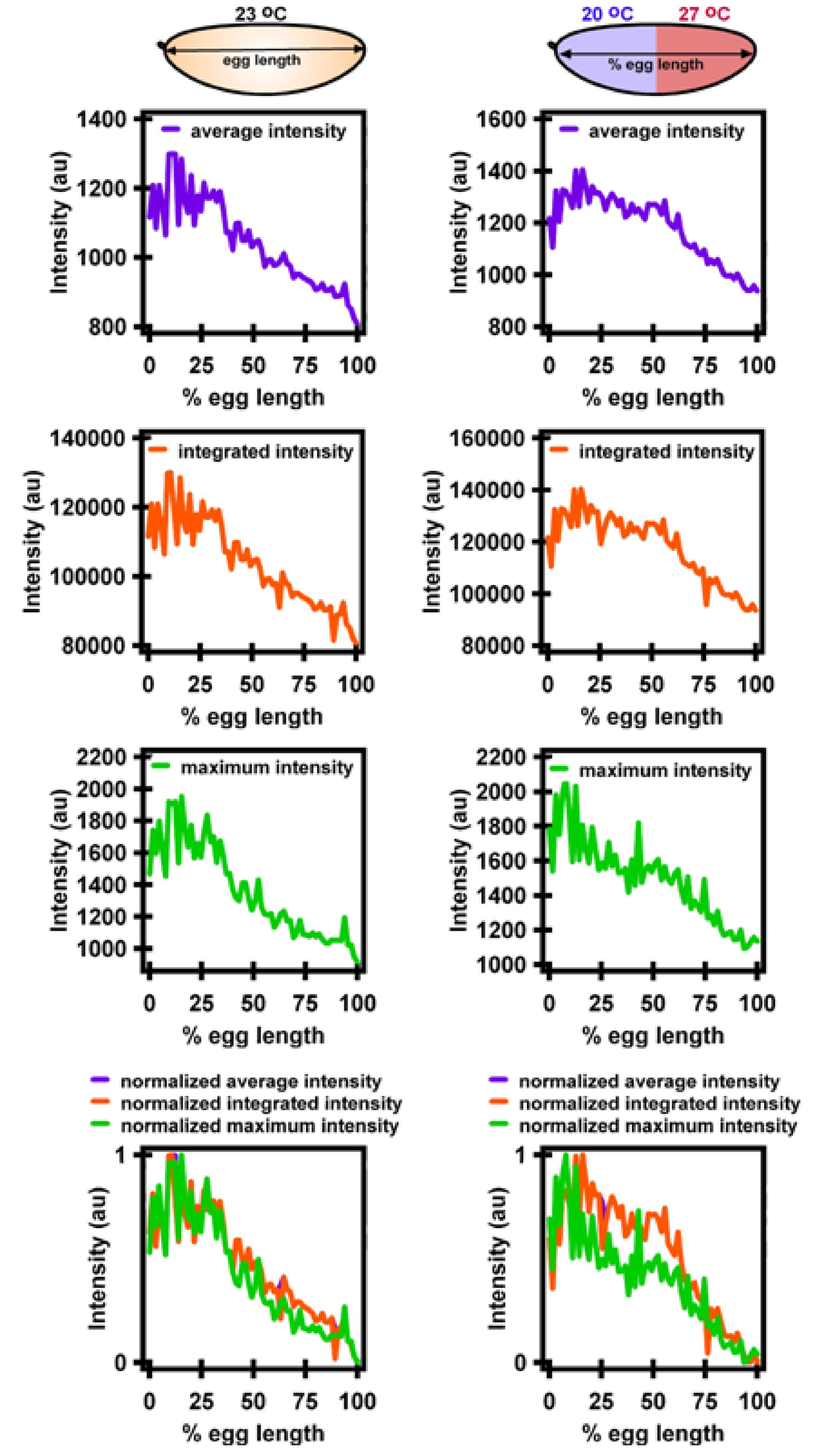

Supplement: Figure S10 — Comparison of Bcd intensity in the embryos shown in Figure 3, measured as an average intensity, integrated intensity or maximum intensity for one time point of the time-series. (14.18 MB TIF) [file pone.0003651.s010.tif]

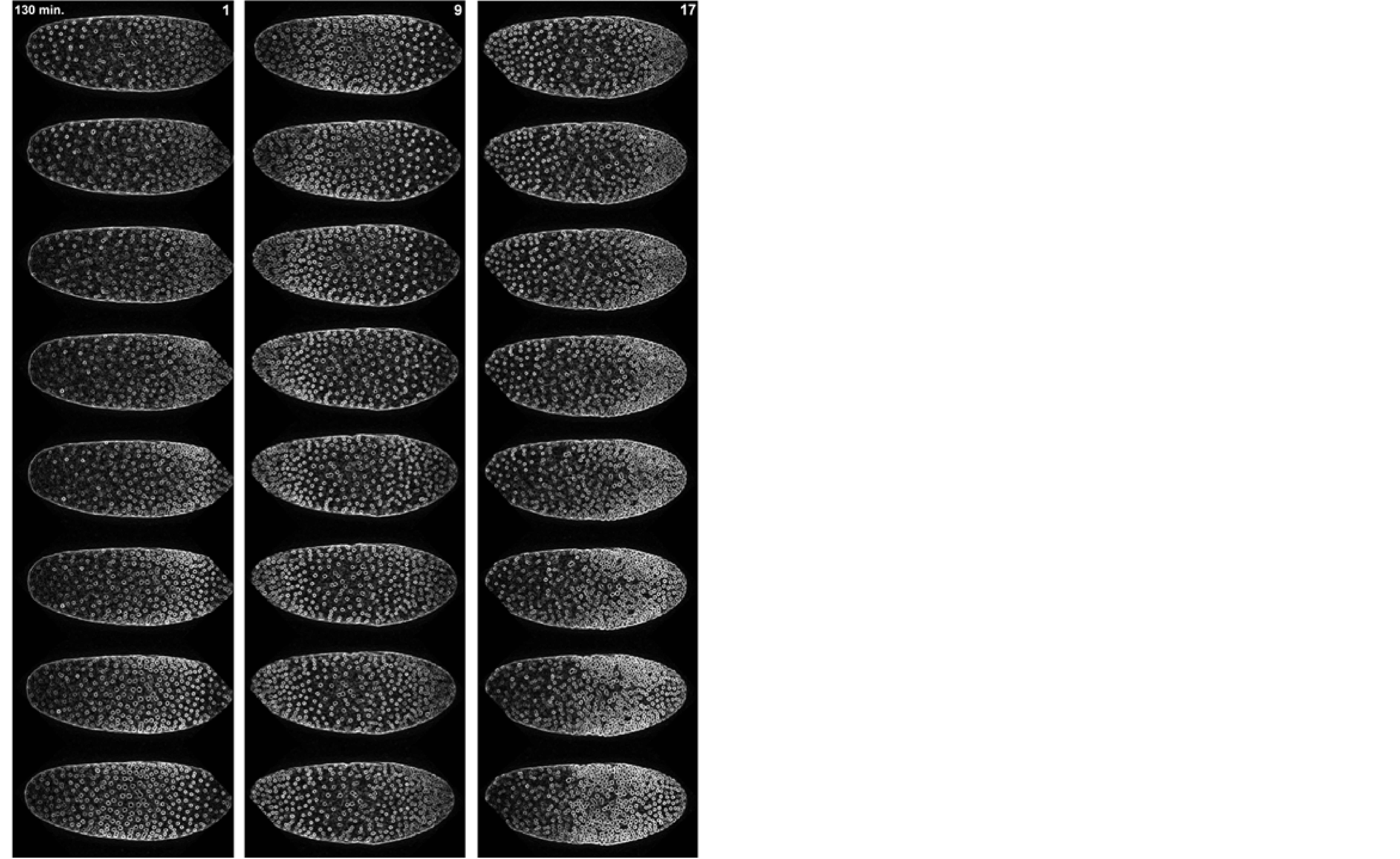

Supplement: Figure S11 — Sobel filter used to track nuclei over time in embryos exposed to a temperature step. (11.16 MB TIF) [file pone.0003651.s011.tif]
